# Sulforaphane enhances the antitumor response of chimeric antigen receptor T cells by regulating PD-1/PD-L1 pathway

**DOI:** 10.1186/s12916-021-02161-8

**Published:** 2021-11-25

**Authors:** Chunyi Shen, Zhen Zhang, Yonggui Tian, Feng Li, Lingxiao Zhou, Wenyi Jiang, Li Yang, Bin Zhang, Liping Wang, Yi Zhang

**Affiliations:** 1 Biotherapy Center, The First Affiliated Hospital of Zhengzhou University, 1 Jianshe East Road, Zhengzhou, 450052 Henan China; 2 Robert H. Lurie Comprehensive Cancer Center, Department of Medicine-Division of Hematology/Oncology, Northwestern University Feinberg School of Medicine, Chicago, IL 60611 USA; 3 Cancer Center, The First Affiliated Hospital of Zhengzhou University, 1 Jianshe East Road, Zhengzhou, 450052 Henan China; 4 School of Life Sciences, Zhengzhou University, Zhengzhou, China; 5 Henan Key Laboratory for Tumor Immunology and Biotherapy, Zhengzhou, China; 6 State Key Laboratory of Esophageal Cancer Prevention & Treatment, Zhengzhou, China

**Keywords:** Sulforaphane, Chimeric antigen receptor T cells, Programmed cell death 1, Programmed cell death ligand 1, Antitumor response

## Abstract

**Background:**

Chimeric antigen receptor T (CAR-T) cell therapy has limited effects in the treatment of solid tumors. Sulforaphane (SFN) is known to play an important role in inhibiting tumor growth, but its effect on CAR-T cells remains unclear. The goal of the current study was to determine whether combined CAR-T cells and SFN could provide antitumor efficacy against solid tumors.

**Methods:**

The effect of combined SFN and CAR-T cells was determined in vitro using a co-culture system and in vivo using a xenograft mouse model. We further validated the effects of combination therapy in patients with cancer.

**Results:**

In vitro, the combination of SFN and CAR-T cells resulted in enhanced cytotoxicity and increased lysis of tumor cells. We found that SFN suppressed programmed cell death 1 (PD-1) expression in CAR-T cells and potentiated antitumor functions in vitro and in vivo. As a ligand of PD-1, programmed cell death ligand 1 (PD-L1) expression was also decreased in tumor cells after SFN treatment. In addition, β-TrCP was increased by SFN, resulting in higher activation of ubiquitination-mediated proteolysis of PD-L1, which induced PD-L1 degradation. The combination of SFN and CAR-T cell therapy acted synergistically to promote better immune responses in vivo compared with monotherapy. In clinical treatments, PD-1 expression was lower, and proinflammatory cytokine levels were higher in patients with various cancers who received CAR-T cells and took SFN orally than that in the control group.

**Conclusion:**

SFN improves the cytotoxicity of CAR-T cells by modulating the PD-1/PD-L1 pathway, which may provide a promising strategy for the combination of SFN with CAR-T cells for cancer immunotherapy.

**Supplementary Information:**

The online version contains supplementary material available at 10.1186/s12916-021-02161-8.

## Background

Chimeric antigen receptor T (CAR-T) cells have been successfully used to treat hematological malignancies [1]. However, patients with solid tumors hardly benefit from CAR-T cell infusion due to several causes, including T cell exhaustion and tumor immune evasion in the tumor microenvironment [2–4]. Tumor infiltrating T cells undergo persistent antigen and inflammation stimulation, resulting in exhaustion, which is characterized by dysfunction and upregulation of multiple inhibitory receptors [5, 6]. Programmed cell death 1 (PD-1) in T cells, a negative immune checkpoint, interacts with programmed cell death ligand 1 (PD-L1) in tumor cells, leading to T cell exhaustion [7, 8]. In the tumor microenvironment, PD-1 expression is sustained highly on exhausted T cells, followed by the diminished production of effector factors, including interferon-gamma (IFN-γ), interleukin-2 (IL-2), and tumor necrosis factor-alpha (TNF-α) [7]. On the other hand, PD-L1 expression on tumor cell surfaces leads to immune escape and poor prognosis [9]. As a multifunctional cytokine, IFN-γ has an antitumor effect and, at the same time, can strongly mediate PD-L1 expression [9–11]. Furthermore, IFN-γ secreted by T cells induces PD-L1 expression on tumor cells and engages with PD-1 on T cells [9, 12]. Thus, T cells lose their effector functions and tumor cells escape from the attack. This evidence establishes a foundation for therapies that target the PD-1/PD-L1 pathway when combined with CAR-T cells. Immune checkpoint blockade based on anti-PD-1/PD-L1 displays a prominent response in tumors with high mutational burdens, including melanoma, lung cancers, and urothelial cancers [13–15]. However, some patients have to discontinue this treatment because of immune-related adverse events, including fatigue, diarrhea, rash, pruritus, endocrinopathies, and serious visceral organ inflammatory toxicities [16, 17]. Hence, whether there is any other safer and more effective drug combination with CAR-T therapies needs to be investigated.

Sulforaphane (SFN) is an isothiocyanate that is enriched in cruciferous vegetables [18]. Since SFN was identified as a phase II enzyme inducer in 1992 [19], various studies have been conducted to reveal its antitumor ability. Epidemiological studies have suggested that diets rich in cruciferous vegetables decrease the risk of cancer incidence [20]. It has been shown that SFN can inhibit the growth of tumor cells and cell cycle progression through multiple mechanisms [21–23]. Studies demonstrated that SFN promotes bladder cancer cell apoptosis and cell circle arrest via ROS production [24]. Furthermore, SFN suppresses breast cancer stem cells by impairing Wnt/β-catenin or NF-κB signaling pathways [25, 26]. Despite deep investigations into tumor cells, their effects can also be observed in immune cells. According to reports, SFN increases the frequency of B and T cells and enhances NK cell activity in a leukemia mouse model [27]. In addition, SFN attenuates the function of Th1/17 by regulating IL-23 and IL-12 production from dendritic cells (DCs) which protects mice from severe autoimmune diseases [28].

Although there are extensive studies, whether and how SFN modulates the tumor microenvironment remains unknown, and whether SFN is available for cancer therapy in the clinic has not been well explored. The exhaustion of CAR-T cells in the tumor microenvironment hinders their antitumor capacity. One of the reasons for this was PD-1/PD-L1 engagement. In this study, we report that SFN downregulated PD-1 expression in CAR-T cells by inhibiting the PI3K/AKT pathway, while SFN promoted PD-L1 degradation in tumor cells by activating the ubiquitination-mediated proteolysis pathway. Furthermore, SFN enhanced the antitumor ability of CAR-T cells both in vitro and in vivo. Our findings revealed the effect of SFN on the antitumor response, which may provide a potential strategy for immunotherapy.

## Methods

### Cell lines

The human lung cancer cell lines A549, H322, and the human embryonic kidney cell line 293 T were purchased from the cell bank of the Chinese Academy of Sciences (Shanghai, China). All cell lines were cultured in DMEM high glucose (Sigma-Aldrich, St. Louis, MO, USA) containing 10% FBS (Lonsera, Uruguay), 100 U/mL penicillin, and 100 μg/mL streptomycin without mycoplasma at 37 °C in a humidified 5% CO_2_ incubator.

### T cell isolation and sorting

Peripheral blood mononuclear cells (PBMCs) from healthy donors were isolated using density gradient centrifugation. CD3^+^ T cells were positively selected using human CD3 microbeads (Miltenyi Biotec, Bergisch Gladbach, Germany) according to the manufacturer’s instructions. The selected CD3^+^ T cells were incubated in RPMI 1640 medium (Sigma-Aldrich, St. Louis, MO, USA) containing 10% FBS, 100 U/mL penicillin, 100 μg/mL streptomycin, and 200 U/mL IL-2 (Beijing SL Pharmaceutical, Beijing, China).

### Generation of meso CAR-T cells

The meso single-chain fragment variable (scFv) was synthesized by Sangon Biotech (Shanghai, China) and cloned into the pCDH lentivirus vector, containing CD3ζ and CD28 activation domains [29]. After activation with anti-CD3/CD28 beads (Miltenyi Biotec, Bergisch Gladbach, Germany), CD3^+^ T cells treated with or without SFN (15 μM; Sigma-Aldrich, St. Louis, MO, USA) were transduced with lentivirus particles encoding meso CAR and cultured with 200 U/mL IL-2 and with or without 15 μM SFN.

### Flow cytometry

FACS Canto II flow cytometer (BD Biosciences, Franklin Lakes, NJ, USA) was used for flow cytometry analysis. Surface marker staining was conducted with fluorescence-conjugated antibodies for 30 min in the dark at 4 °C. For intracellular staining, the cells were stained with antibodies against intracellular cytokines after surface antibody staining, fixation, and permeabilization. All antibodies were purchased from BioLegend (San Diego, CA, USA): APC/Cyanine7 anti-human CD8 (SK1), PE anti-human CD107a (H4A3), PE anti-human PD-1 (EH12.2H7), APC anti-human Tim-3 (F38-2E2), PE anti-human CD69 (FN50), PE anti-human PD-L1 (29E.2A3), APC anti-human IFN-γ (4S.B3), PE anti-human IL-2 (MQ1-17H12), APC anti-human Perforin (dG9), and PE anti-human/mouse Granzyme B Recombinant (QA16A02), except PE anti-human mesothelin (REA1057; Miltenyi Biotec, Bergisch Gladbach, Germany).

### Specific lysis analysis

Tumor cells were incubated with CAR-T cells at different effector to target (E:T) ratios for 6 h. Tumor cells were collected and stained with Annexin-V (BioLegend, San Diego, CA, USA) in Annexin-V Binding Buffer (BioLegend, San Diego, CA, USA) for 15 min in the dark. Flow cytometry was used for analysis after the addition of propidium iodide (BioLegend, San Diego, CA, USA).

### Western blot analysis

To collect whole-cell lysates, cells were lysed in RIPA lysis buffer supplemented with a Protease Inhibitor Cocktail (Sigma-Aldrich, St. Louis, MO, USA), and the cell lysates were centrifuged at 12,000 rpm. The supernatant was collected, mixed with loading buffer, and boiled for 15 min to disengage the protein secondary structure. The mixture was resolved on SDS-PAGE gels (BioSci™ NewFlash Protein AnyKD PAGE; DAKEWE, Beijing, China) and then transferred onto nitrocellulose membranes (GE life, Pittsburgh, PA, USA). After blocking with 5% non-fat milk, the membranes were incubated with primary antibodies overnight at 4 °C. The bands were probed with the appropriate secondary antibodies and detected by enhanced chemiluminescence. The following primary antibodies were purchased from Cell Signaling Technology (Danvers, MA, USA): PD-1 (D4W2J, 1:1000), phospho-AKT (Ser473) (D9E, 1:2000), phospho-mTOR (Ser2448) (D9C2, 1:1000), phospho-S6 ribosomal protein (Ser235/236) (D57.2.2E, 1:2000), AKT (pan) (C67E7, 1:1000), mTOR (7C10, 1:1000), S6 Ribosomal Protein (54D2, 1:1000), β-TrCP (D13F10, 1:1000), and β-Actin (8H10D10, 1:1000), except anti-PD-L1 antibody [EPR19759] (ab213524; Abcam, UK, 1:1000).

### RNA sequencing

We first purified and enriched the RNA from samples. For mRNA enrichment, RNA with a polyA tail was enriched using magnetic beads with Oligo-dT. For RNA purification, DNA probes were used to hybridize the rRNA. The hybridized products were subsequently degraded by RNase H and DNase I. Next, the RNA was shredded into fragments and was reverse-transcribed to cDNA using random N6 primers. The ends of the synthesized double-stranded DNA were filled, the 5 'ends were phosphorylated, and the ligation products were amplified by PCR with specific primers. After thermal denaturation into a single strand, the PCR products were circularized with a bridge primer to obtain a single-stranded circular DNA library. Finally, the BGISEQ-500 platform was used for high-throughput sequencing.

### Gene Set Enrichment Analysis (GSEA)

Whole gene counts from RNA-seq data were selected for the GSEA enrichment analysis [30]. “c2.cp.kegg.v6.0.symbol” from the MSigDB database was selected to perform pathway enrichment based on the KEGG database. Groups were consistent with RNA-seq, and other parameters were performed as default. Gene expression files were modified as software’s request.

### Quantitative real-time PCR

Total RNA was extracted with TRIzol (Invitrogen, Carlsbad, CA, USA), and the quality and concentration were detected using a NanoDrop 2000 spectrophotometer (Thermo Fisher Scientific, Waltham, MA, USA). cDNA was synthesized using the Primescript RT Reagent Kit (TakaRa, Dalian, China). Quantitative real-time PCR (qRT-PCR) analysis was performed using the SYBR Green Master Mix (Roche, Basel, Switzerland). The primers used are shown in Additional file 1: Table S1.

### Tumor cell transfection

A549 or H322 cells (1.5 × 10^5^) were seeded in 6-well plates. Three *BTRC* short interfering RNA (siRNA) or scramble (siScr) sequences (Shanghai Gene-Pharma, Shanghai, China) were transfected into cells using jetPRIME transfection reagent (Polyplus transfection, France) according to the manufacturer’s protocol. After 48 h, transfection efficacy was tested by qRT-PCR and western blot.

### In vivo studies

All mouse experiments were approved by the Institutional Animal Care and Use Committee of the First Affiliated Hospital of Zhengzhou University. Female 5-week-old NOD-SCID mice were purchased from Beijing Vital River Laboratory Animal Technology Company and were fed in the Henan Key Laboratory for Pharmacology of Liver Diseases. Luciferase-expressing H322 (H322-luc) cells (5 × 10^5^) were injected subcutaneously (s.c.) into mice. Five days later, mice received 5 × 10^6^ meso CAR-T cells intravenously injection (i.v.) with SFN (LKT Labs, St. Paul, MN, USA) or anti-PD-1 (Pembrolizumab Injection, Keytruda, MSD, USA) treatment. Tumor growth was measured by bioluminescent imaging using a Xenogen IVIS-200 Spectrum camera and Living Image version 4.4 (Caliper Life Sciences, Waltham, MA, USA) for image acquisition and analysis. For the analysis of tumor- and spleen-infiltrating meso CAR-T cells, 7 days after meso CAR-T cell injection, cells from fresh tumor tissues were harvested using a tumor dissociation kit (Miltenyi Biotec, Bergisch Gladbach, Germany) according to the manufacturer’s instructions, while the spleens were ground directly for cell collection. Phenotypes and cytokine secretion were evaluated using flow cytometry. Mice were sacrificed when the values of total flux were over 2 × 10^11^.

### Immunohistochemistry (IHC)

Tissue sections from xenograft tumor were first deparaffinized. The sections were placed in 0.01 M sodium citrate buffer for antigen retrieval. Then, 3% H_2_O_2_ was added to remove endogenous peroxidase and 5% normal goat serum was used to block nonspecific binding. Then, the sections were incubated with the primary antibody (anti-PD-L1 antibody [PDL1/2746] (ab238697; Abcam, UK)) overnight at 4 °C, followed by incubation with horseradish peroxidase-linked secondary antibody at room temperature for 30 min. After stained with the DAB solution, the samples were counterstained with hematoxylin, dehydrated, and sealed with neutral gum.

PD-L1 expression was evaluated by the immunohistochemical scoring (IHS). The proportion of positive cells was scored as follows: 0 (no positive cells), 1 (1–25%), 2 (26–50%), 3 (51–75%), and 4 (76–100%).The staining intensity was scored on a 0 to 3 scale (0, negative; 1, weak; 2, moderate; 3, strong). The final score was based on the addition of these two scores: negative (−, 0), weakly positive (+, 1–3), moderately positive (++, 4–5), and strongly positive (+++, 6–7).

### Human Subjects

Peripheral blood was obtained from patients with cancer who voluntarily participated in a clinical trial approved by the Institutional Review Board of the First Affiliated Hospital of Zhengzhou University (NCT03229876, NCT03638206). Written informed consent was obtained from all the participants. Four patients received sulforaphane from broccoli sprout extract (Swanson, USA), two capsules per day for 30 days at the beginning of CAR-T therapy, and another four patients only received CAR-T therapy as a control group. The clinical characteristics of the patients are presented in Additional file 2: Table S2. PBMCs from the peripheral blood of these patients were collected by density gradient centrifugation. PD-1 expression and cytokine secretion were analyzed by flow cytometry.

### Statistical analysis

Data were analyzed using GraphPad Prism 8.2.1(GrapPad Software, La Jolla, CA, USA) and R language (version 3.6). Student’s *t* test was used for comparison of two groups. Both methods were achieved by the “Analyze” function in GraphPad Prism. Survival curves were determined by Kaplan-Meier analysis using the R packages “Survival” and “Survminer.” All boxplot and barplot images are presented as the mean ± SEM. Statistical significance is shown as *(*P* < 0.05), **(*P* < 0.01), ***(*P* < 0.001), and **** (*P* < 0.0001), and ns meant not significant (*P* > 0.05).

## Results

### SFN promotes cytotoxicity of meso CAR-T cells in vitro by downregulating PD-1 expression

**Fig. 1:**
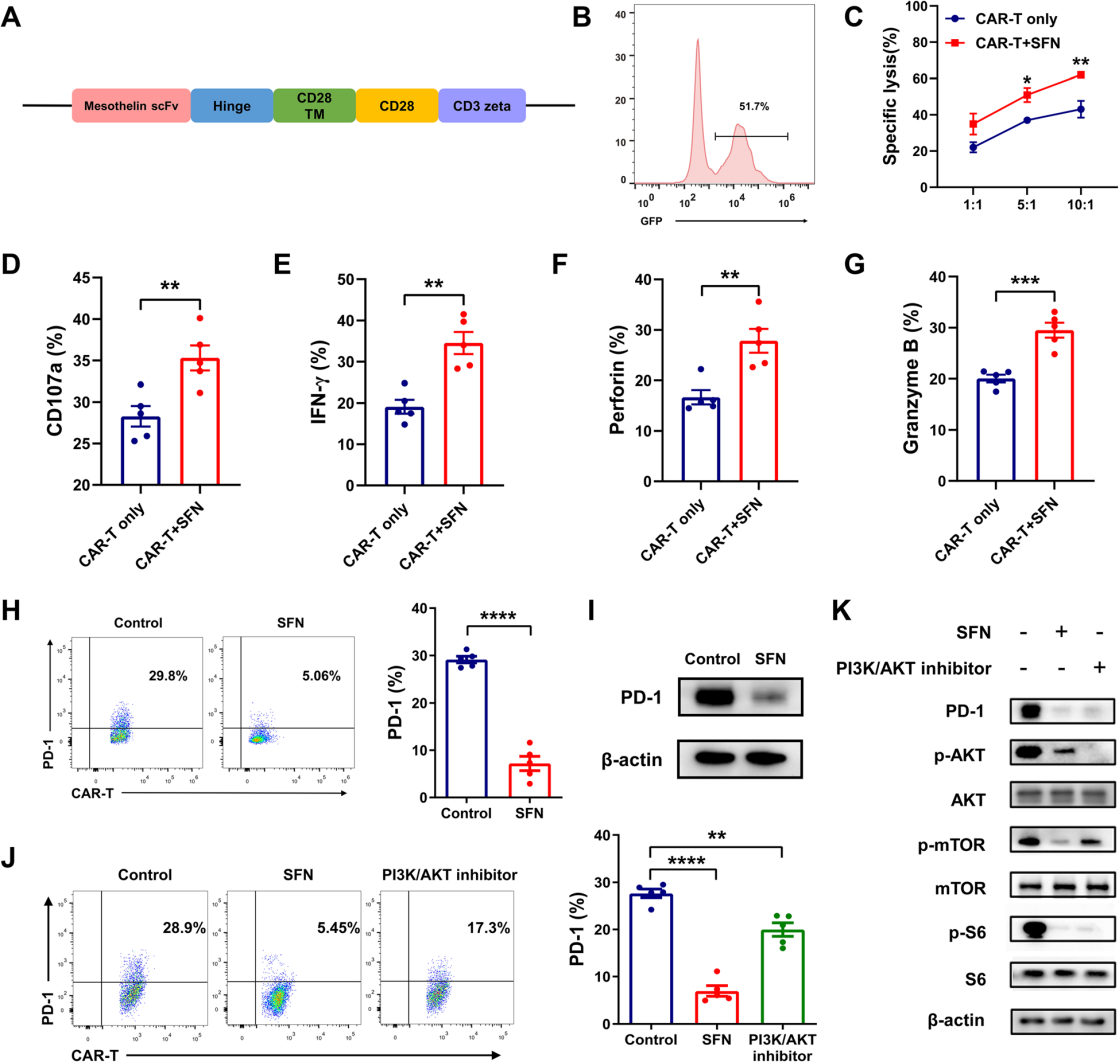
Sulforaphane (SFN) enhanced the antitumor function of meso CAR-T cells in vitro by downregulating PD-1 expression. **A** Schematics of meso CAR constructs. **B** Transduction efficacy of meso CAR-T cells. Transduction efficacy was examined by green fluorescent protein (GFP) expression on day 5. **C** Analysis of specific lysis of tumor cells. H322 cells were incubated with meso CAR-T cells at various effector to target (E:T) ratios (1:1, 5:1, and 10:1) for 6 h, and lysis of H322 cells was analyzed by flow cytometry. **D** H322 cells were incubated with meso CAR-T cells at a 10:1 ratio for 6 h. Expression of CD107a on CAR-T cells was tested by flow cytometry. **E–G** After co-culturing with H322 cells at a 1:1 ratio for 24 h, the secretion of IFN-γ (**E**), perforin (**F**), and granzyme B (**G**) by CD8^+^ meso CAR-T cells was investigated by flow cytometry. **H, I** PD-1 expression on CD8^+^ meso CAR-T cells with or without SFN treatment was analyzed by flow cytometry (**H**) and western blot (**I**). **J** PD-1 expression on CD8^+^ meso CAR-T cells was tested by flow cytometry, with or without treatment with SFN and PI3K/AKT inhibitor PF-04691502 (10 μM). **K** Western blot analysis of phosphorylated and total AKT, mTOR, and S6 proteins, as well as PD-1 and β-actin levels, in meso CAR-T cells with or without SFN and PF-04691502 treatment. Results are representative of five independent experiments. *, *P* < 0.05; **, *P* < 0.01; ***, *P* < 0.001; ****, *P* < 0.0001 (Student’s *t* test)

We transduced CD3^+^ T cells with a second-generation anti-mesothelin CAR construct containing the CD3ζ and CD28 activation domains (Fig. 1A). The transduction efficacy was greater than 51% (Fig. 1B). Surface mesothelin expression was evaluated in lung tumor cell lines by flow cytometry. H322 cells highly expressed mesothelin with a percentage of over 90% (data not shown). Therefore, we used H322 as the target cells. To further analyze the role of SFN in the antigen-specific cytotoxicity of meso CAR-T cells, the tumor cells were incubated with meso CAR-T cells in the presence or absence of SFN. Our results showed that meso CAR-T cells had a more potent cytotoxic function against tumor cells with addition of SFN (Fig. 1C) and expressed higher levels of CD107a as an indicator of degranulation (Fig. 1D). Meanwhile, the cytokine secretion of IFN- γ, perforin, and granzyme B was significantly increased in the SFN-treated CAR-T cell group compared to the control group (Fig. 1E–G). However, whether SFN could prevent CAR-T cell exhaustion remains unknown. We then tested the effect of SFN on PD-1 expression and found that PD-1 was significantly inhibited by SFN in meso CAR-T cells (Fig. 1H, I, Additional file 3: Fig. S1A). It has been reported that the PI3K/AKT signaling pathway contributes to many aspects of T cell differentiation, metabolism, function, and survival [31]. Thus, we determined whether SFN affected the PI3K/AKT axis. We treated meso CAR-T cells with SFN or PI3K inhibitor PF-04691502 and observed that PD-1 was downregulated both in SFN and PF-04691502-treated meso CAR-T cells (Fig. 1J). Meanwhile, SFN inhibited the downstream cellular activity of PI3K similar to PF-04691502, including p-AKT, p-mTOR, and p-S6, which was consistent with PD-1 reduction (Fig. 1K, Additional file 3: Fig. S1A). However, we found that the effect of the PI3K inhibitor on PD-1 was weaker than that of SFN, which indicated that the PI3K/AKT axis was not the only pathway for SFN. Our findings suggest that SFN may regulate PD-1 expression partially through a PI3K/AKT-dependent pathway.

### Meso CAR-T cells show stronger antitumor efficacy in vivo after SFN treatment

**Fig. 2:**
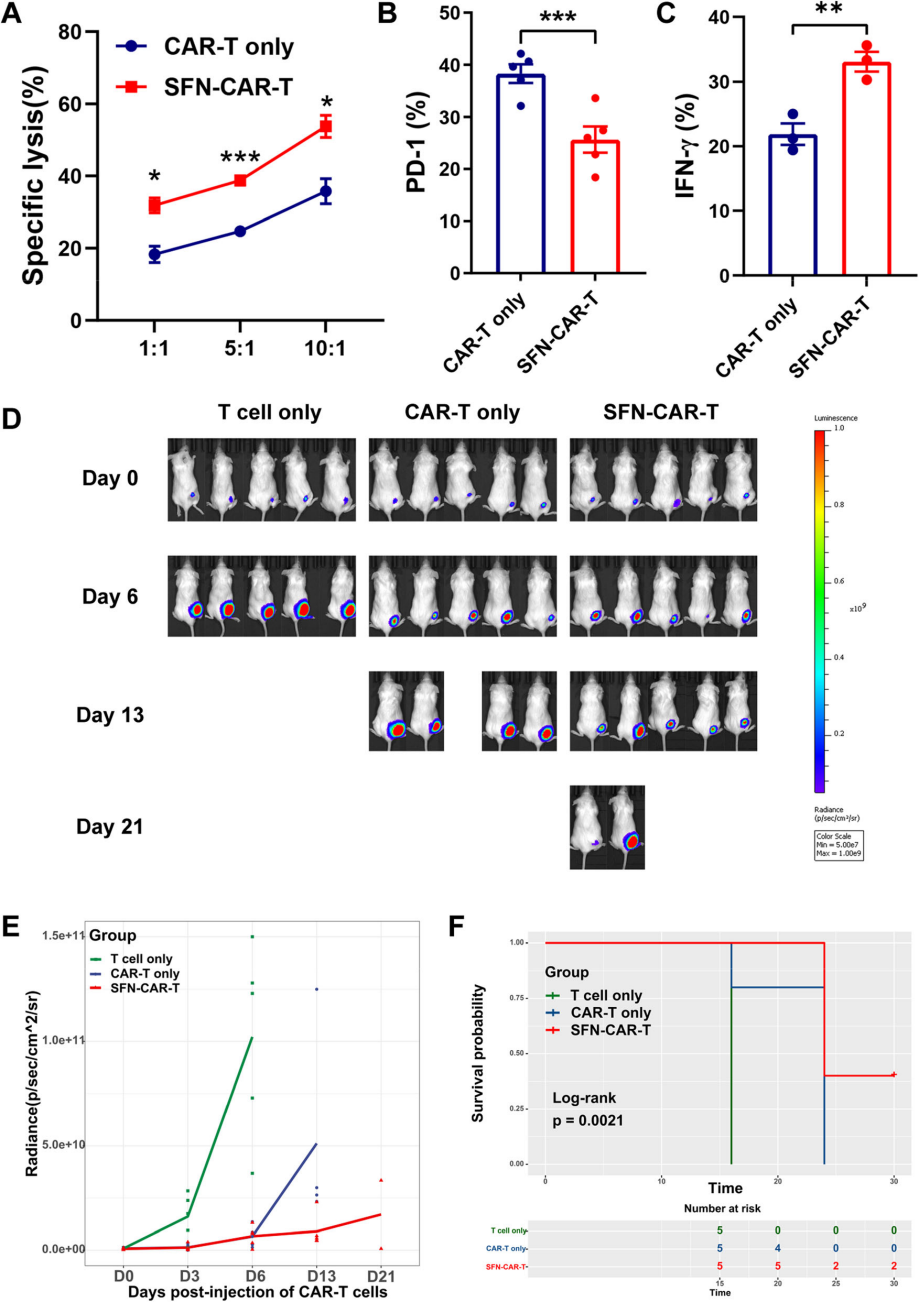
CAR-T cells showed stronger antitumor activity in vivo after SFN pre-treatment. **A** Analysis of specific lysis of tumor cells. H322 cells were incubated with meso CAR-T cells at various effector to target (E:T) ratios (1:1, 5:1, and 10:1) for 6 h, and lysis of H322 cells was analyzed by flow cytometry. **B** The expression of PD-1 on SFN-treated CD8^+^ meso CAR-T cells was analyzed by flow cytometry. **C** After co-culturing with H322 cells at a 1:1 ratio for 24 h, the secretion of IFN-γ by CD8^+^ meso CAR-T cells was investigated by flow cytometry. **D, E** NOD/SCID mice were injected with 5 × 10^5^ H322-luc cells. After 5 days, mice were divided into three groups randomly and were infused with 5 × 10^6^ T cells (i.v.). Bioluminescence images of five representative mice in the three treatment groups are shown after adoptive therapies (**D**). The bioluminescence signal was measured at different time points (**E**). **F** Kaplan-Meier survival curves are presented. *, *P* < 0.05; ***, *P* < 0.001; ****, *P* < 0.0001 (Student’s *t* test)

Next, we analyzed the function of SFN-treated meso CAR-T cells in vivo. H322-luc cells were injected subcutaneously into mice. The meso CAR-T cells were treated with SFN in vitro before adoptive transfer. Compared with untreated meso CAR-T cells, SFN-treated meso CAR-T cells had a more potent cytotoxic function against tumor cells (Fig. 2A). The expression of the inhibitory receptors PD-1 in SFN-treated meso CAR-T cells were attenuated that of the IFN-γ secretion was increased (Fig. 2B, C). Then, the untransduced T cells (T cell only), meso CAR-T cells (CAR-T only), and SFN-treated meso CAR-T cells (SFN-CAR-T) were infused i.v. into tumor-bearing mice. Tumor growth in the mouse models was assessed using a bioluminescence (IVIS) imaging system. Compared with the CAR-T-only group, the SFN-CAR-T group showed a reduction in tumor growth and improvement in overall survival (Fig. 2D–F). These observations support that SFN improves the meso CAR-T cytotoxicity function in vivo.

### SFN downregulates IFN-γ-induced PD-L1 expression on tumor cells

**Fig. 3:**
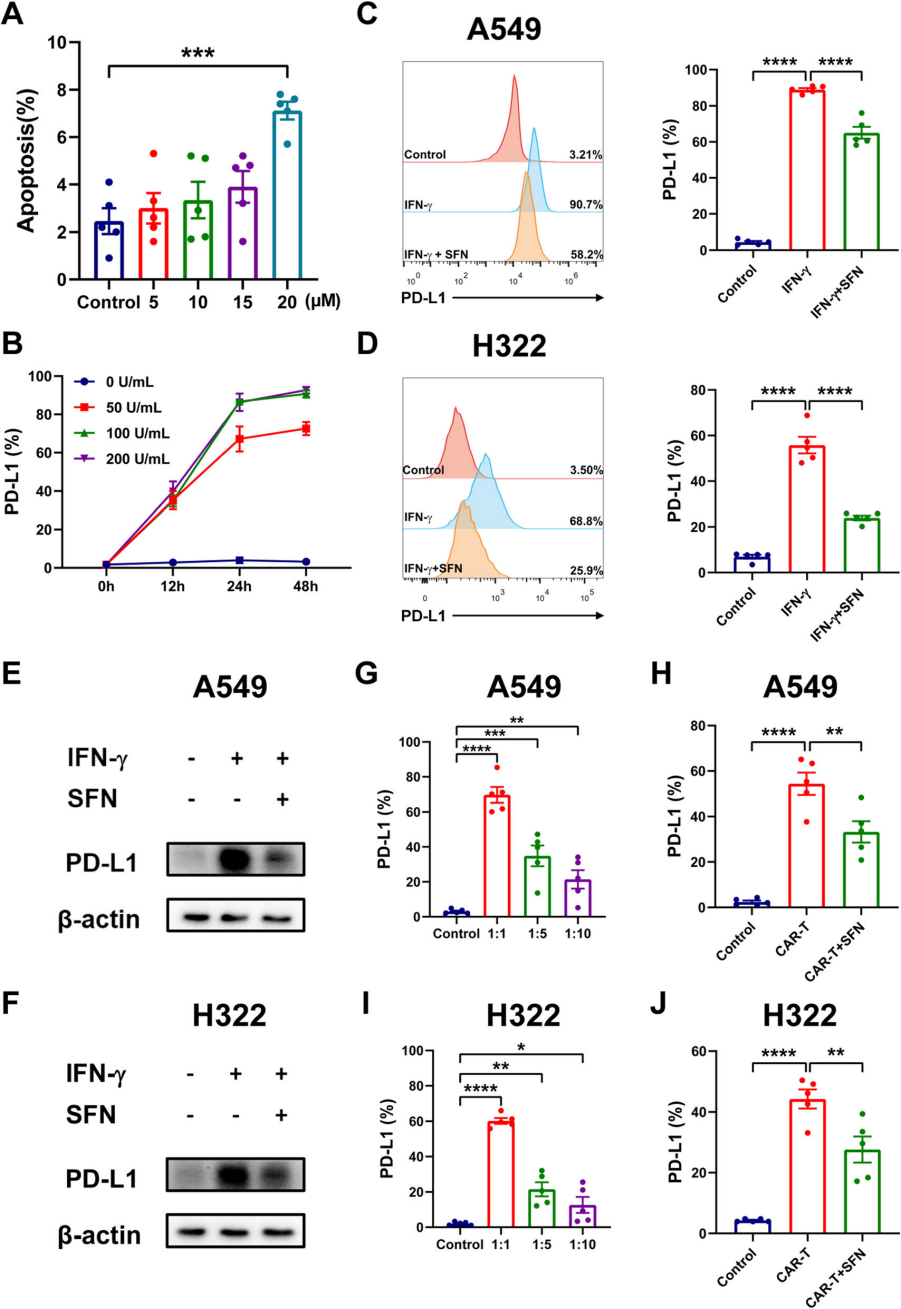
SFN attenuated IFN-γ-induced PD-L1 expression on tumor cells. **A** A549 cells were treated with SFN at different concentrations. After 24 h, the cells were collected and stained with Annexin-V in a binding buffer. Then, apoptosis was tested by flow cytometry after the addition of propidium iodide. **B** A549 cells were treated with IFN-γ at various concentrations for different time periods. PD-L1 expression was investigated by flow cytometry. **C**, **D** A549 and H322 cells were treated with or without IFN-γ and SFN for 24 h, and PD-L1 expression was tested by flow cytometry. **E, F** Western blot was performed to detect PD-L1 expression after the cells were treated with IFN-γ or SFN. **G–J** A549 and H322 cells were incubated with meso CAR-T cells at various effector to target (E:T) ratios (1:1, 1:5, 1:10) for 24 h, and PD-L1 expression on tumor cells was examined by flow cytometry (**G**,**I**). A549 and H322 cells were incubated with meso CAR-T cells at a 1:1 ratio and were additionally treated with SFN. After 24 h, PD-L1 expression was tested by flow cytometry (**H**, **J**). Results are representative of five independent experiments. *, *P* < 0.05; **, *P* < 0.01; ***, *P* < 0.001; ****, *P* < 0.0001 (Student’s *t* test)

PD-L1 is a ligand of PD-1 that is expressed on tumor cells. In previous reports, SFN had a direct inhibitory effect on tumor cells [20]; however, whether SFN can inhibit PD-L1 expression remains unclear. To examine the effect of SFN on tumor cells besides direct inhibition of cell growth, we treated tumor cells with 15 μM SFN which had little impact on tumor apoptosis (Fig. 3A). IFN-γ is known to be the most potent inducer of PD-L1. We found that PD-L1 expression was induced by IFN-γ in a dose- and time-dependent manner (Fig. 3B). Thus, we used 100 U/mL IFN-γ to treat tumor cells for 24 h in subsequent studies. Furthermore, the results from both flow cytometry and western blot analysis demonstrated that the addition of IFN-γ strongly mediated the expression levels of PD-L1 on A549 and H322 cells, while SFN significantly reduced this upregulation in both cell lines (Fig. 3C–F, Additional file 3: Fig. S1B). Similar results were observed in tumor cells co-cultured with meso CAR-T cells. PD-L1 expression, which was induced by meso CAR-T cells, was downregulated in the presence of SFN (Fig. 3G–J). These results revealed that SFN significantly attenuated the IFN-γ-induced PD-L1 expression on tumor cells.

### SFN negatively regulates PD-L1 degradation in a poly-ubiquitination dependent manner

**Fig. 4:**
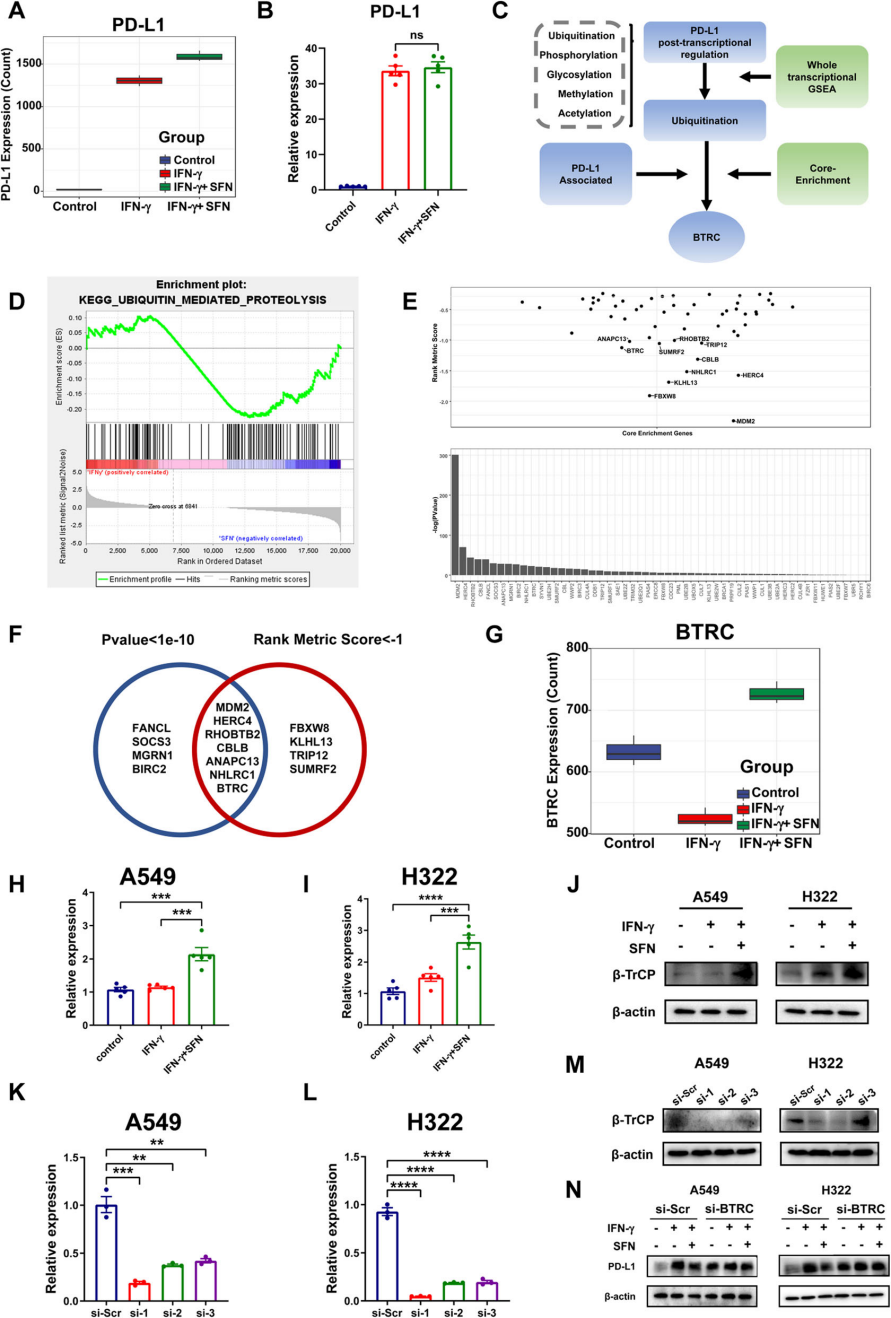
PD-L1 was downregulated by SFN in a poly-ubiquitination dependent manner. **A** PD-L1 expression (count) according to RNA-seq data. **B** Analysis of PD-L1 expression on A549 cells at the transcriptional level in three groups (control, IFN-γ, and IFN-γ + SFN) by qRT-PCR. **C** Schematic representation of the bioinformatics analysis process. **D** Gene set enrichment analysis (GSEA) analysis of whole-gene counts from RNA-seq data. **E** The core enrichment genes enriched in ubiquitination-mediated proteolysis pathway were ranked by Rank Metric Score and *p* value. **F** List of genes with scores less than − 1 and *p* values less than 1e−10. **G** Analysis of *BTRC* expression (count) from RNA-seq data. **H–J** Analysis of *BTRC* (β-TrCP) expression in the control, IFN-γ, and IFN-γ + SFN groups by qRT-PCR and western blot in A549 and H322 cells. **K–M** Analysis of the transfection efficacy of *BTRC* siRNA in A549 and H322 cells by qRT-PCR and western blot. **N** PD-L1 expression was detected in the presence of IFN-γ or SFN after transfection with si-Scr or si-BTRC by western blot on A549 and H322 cells. *, *P* < 0.05; **, *P* < 0.01; ***, *P* < 0.001; ****, *P* < 0.0001; ns, not significant (Student’s *t* test)

To further investigate the molecular mechanisms of PD-L1 downregulation by SFN, A549 cells were treated with different treatments for RNA sequencing (RNA-seq), including untreated group (control), IFN-γ-treated group (IFN- γ) and IFN- γ together with SFN-treated group (IFN- γ + SFN). Interestingly, PD-L1 mRNA expression was not consistent with the protein level, which showed no significant difference between the IFN-γ and IFN-γ + SFN groups. Furthermore, this result was confirmed in our samples (Fig. 4A, B), indicating that SFN may regulate PD-L1 expression by inducing post-translational modifications (PTMs). The bioinformatics analysis process is shown in Fig. 4C.

According to GSEA analysis, we found that the ubiquitination-mediated proteolysis pathway was highly activated in the presence of SFN (Fig. 4D). We then analyzed the core enrichment genes enriched in this pathway and ranked them according to their Rank Matrix Score. Genes with a score less than − 1 were identified as key genes that were actively involved in the ubiquitination pathway in the IFN-γ + SFN group. Moreover, we calculated the fold change and *p* values of all the core enrichment genes between the IFN-γ and IFN-γ + SFN groups (Fig. 4E). After taking the intersection of genes with scores less than − 1 and *p* values less than 1e−10, we obtained seven genes (*MDM2*, *HERC4*, *RHOBTB2*, *CBLB*, *ANAPC13*, *NHLRC1*, *BTRC*) (Fig. 4F). Among these genes, *BTRC*, called β-TrCP at the protein level, is characterized as a ubiquitin E3 ligase adaptor protein that mediates PD-L1 poly-ubiquitination and degradation [32]. Expression of *BTRC* was significantly upregulated by SFN (Fig. 4G). We then verified *BTRC* expression in A549 and H322 cells by qRT-PCR and western blot (Fig. 4H–J, Additional file 3: Fig. S1C). Additionally, we silenced *BTRC* expression using siRNA (Fig. 4K–M, Additional file 3: Fig. S1D). Thus, the si-1 and si-Scr sequences were used in subsequent studies. Due to *BTRC* knockdown, the expression of PD-L1 was not downregulated by SFN (Fig. 4N, Additional file 3: Fig. S1E). These data demonstrated that SFN suppressed PD-L1 by promoting the ubiquitination process by increasing *BTRC* expression.

### SFN and meso CAR-T cells synergistically mediate tumor remission in vivo

**Fig. 5:**
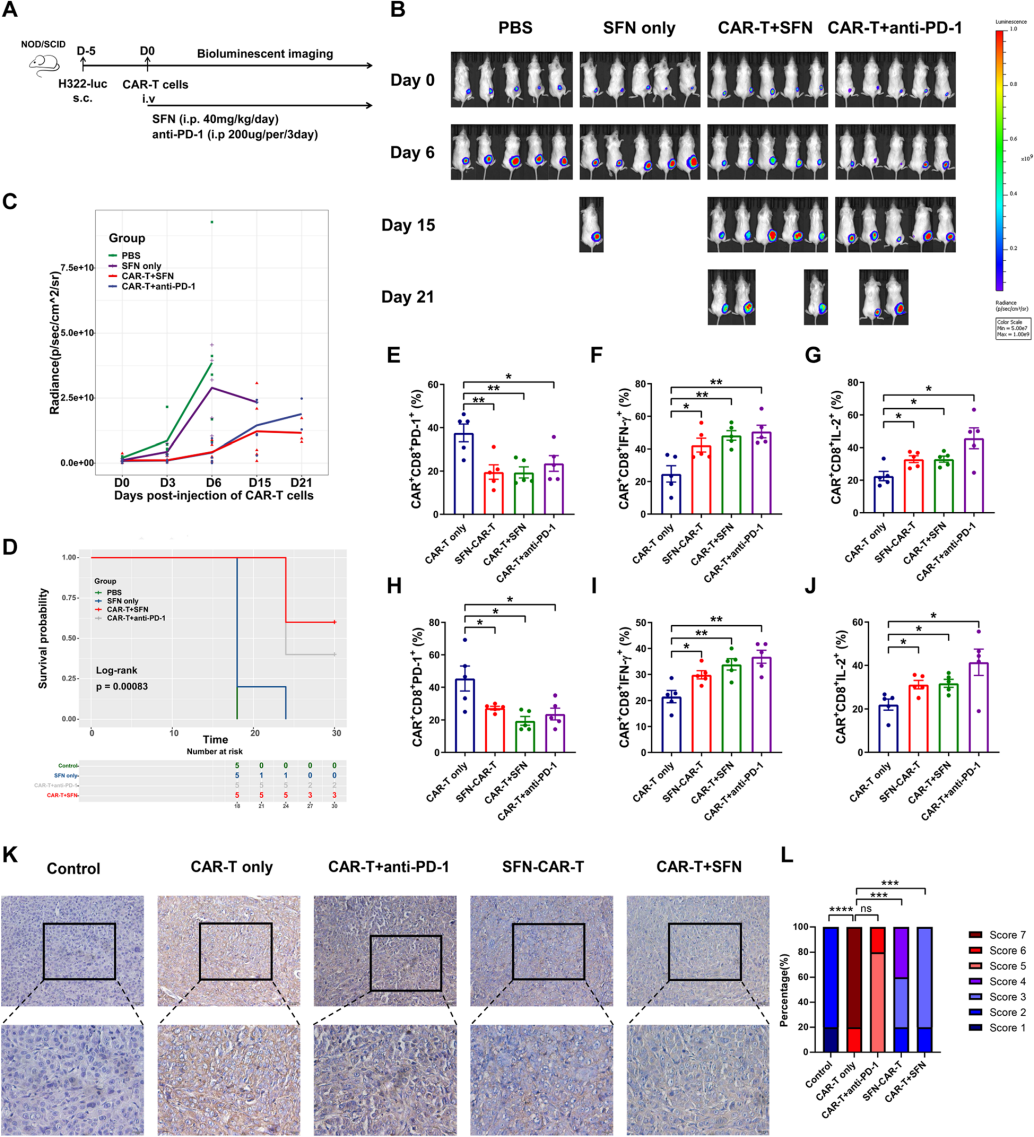
SFN and meso CAR-T cells act synergistically to mediate tumor regression in vivo. **A** H322-luc cells (5 × 10^5^) were injected s.c. into NOD/SCID mice. Five days later, mice were divided into four groups randomly. Two groups were treated with PBS or SFN (40 mg/kg/day, i.p.). Another two groups received 5 × 10^6^ meso CAR-T cell infusion (i.v.) combined with SFN (40 mg/kg/day) or anti-PD-1 (200 μg/mouse/3 days) injection (i.p). **B, C** Bioluminescence images of five representative mice in the four treatment groups are shown after adoptive therapies (**B**). The bioluminescence signal was measured at different time points (**C**). **D** Survival analysis was performed by using Kaplan-Meier survival curves. **E–J** NOD/SCID mice were injected with 5 × 10^5^ H322-luc cells. Five days later, mice were injected (i.v.) with meso CAR-T cells only (CAR-T only), meso CAR-T cells pre-treated with SFN (SFN-CAR-T), a combination of meso CAR-T cells and SFN (40 mg/kg/day, i.p.) (CAR-T + SFN), and meso CAR-T cells combined with anti-PD-1 (200 μg/mouse/3 days, i.p.) (CAR-T + anti-PD-1). After 7 days, tumor tissues and spleens were harvested. Then, PD-1 expression and secretion of cytokines including IFN-γ and IL-2 of CD8^+^ meso CAR-T cells from tumor tissues (E–G) and spleens (H–J) were tested by flow cytometry. **K** Representative images of PD-L1 immunohistochemical staining on tumor tissues from different groups of mice model. **J** IHC score of PD-L1 in tumor tissues. *, *P* < 0.05; **, *P* < 0.01; ***, *P* < 0.001; ****, *P* < 0.0001 ns; not significant (Student’s *t* test)

We investigated the effect of meso CAR-T cells combined with SFN against tumors in our established xenograft mouse model. Tumor-bearing mice received a daily intraperitoneal injection (i.p.) of SFN only (SFN only), or meso CAR-T cell infusion with exogenous SFN administration (CAR-T + SFN), or anti-PD-1 treatment (CAR-T + anti-PD-1) (Fig. 5A). Although SFN injection alone did not induce complete tumor regression, a single meso CAR-T adoptive transfer synergy with SFN showed a stronger ability to delay tumor growth and prolonged survival, which was similar to the effect in conjunction with anti-PD-1 therapy (Fig. 5B–D). To estimate the impact of different modes of administration of SFN on meso CAR-T cells in vivo, we tested PD-1 expression and cytokine secretion of tumor- and spleen-infiltrating meso CAR-T cells from mice with different treatments (Fig. 5E–J). Regardless of the tumor tissue or spleen, CD8^+^ meso CAR-T cells with SFN pre-treatment or exogenous SFN administration showed less PD-1 expression and stronger IFN-γ and IL-2 secretion ability compared with untreated meso CAR-T cells, while exhibiting no significance compared to treatment with meso CAR-T cells plus anti-PD-1. To verify the impact of SFN on PD-L1 in vivo, we analyzed the PD-L1 expression on tumor tissues by IHC staining (Fig. 5K, L). The results showed that PD-L1 expression was significantly decreased after SFN treatment. Overall, our results indicated that different SFN administrations enhanced the function of meso CAR-T cells.

### SFN improves antitumor ability of CAR-T cells in patients with cancer

**Fig. 6:**
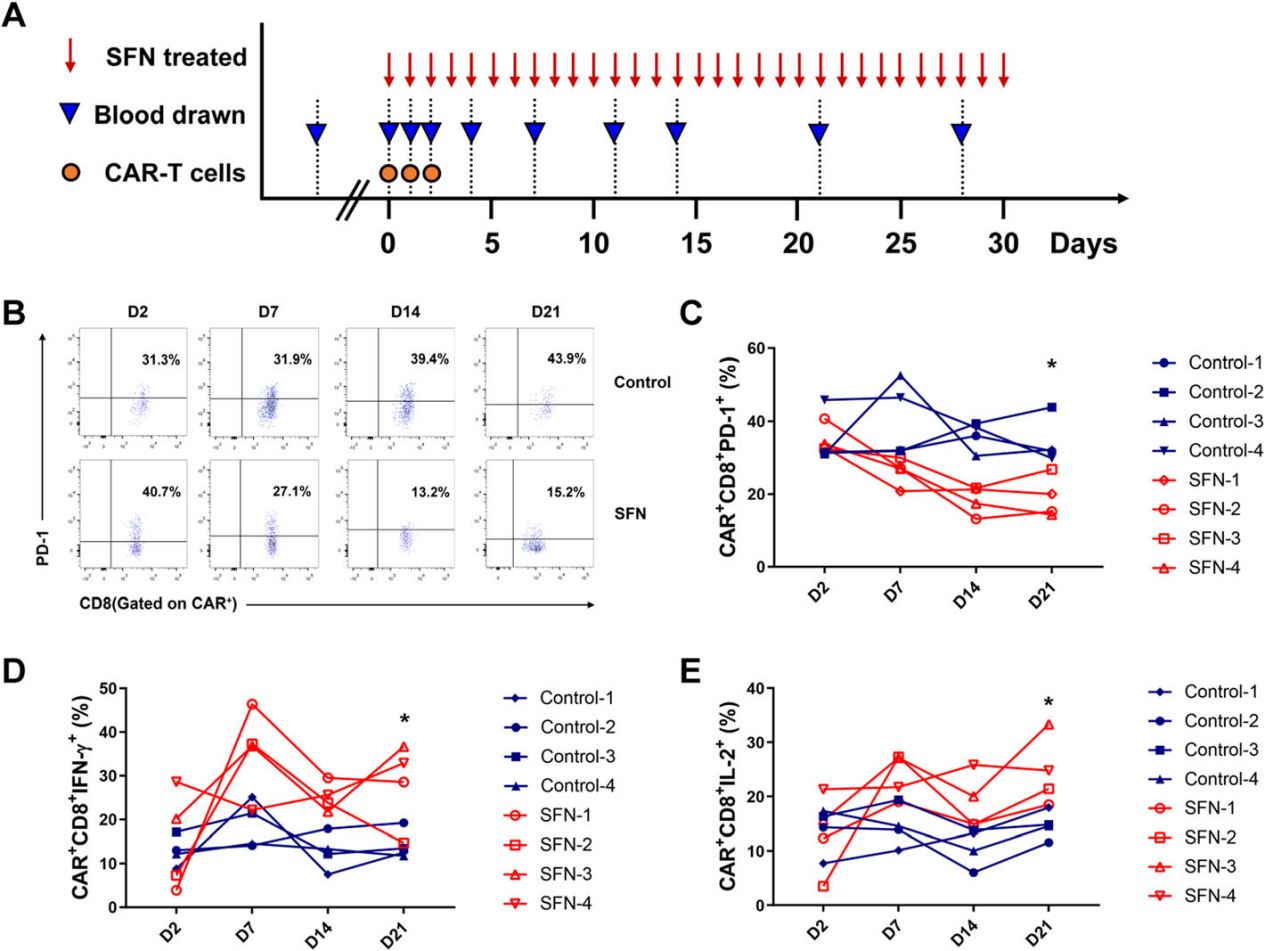
SFN enhances the antitumor ability of CAR-T cells in patients with cancer. **A** Time line of immunotherapy treatment. **B** The expression of PD-1 on CD8^+^ CAR-T cells derived from patients who had or had not received sulforaphane (SFN) at different time points. **D**, **E** The expression of IFN-γ and IL-2 in CD8^+^ CAR-T cells derived from patients who had or had not received SFN on day 2, 7, 14, and 21. *, *P* < 0.05; **, *P* < 0.01 (Student’s *t* test)

To determine whether SFN could promote CAR-T cell cytotoxicity in patients with cancer who received adoptive immunotherapy, we simultaneously treated patients with SFN orally and examined PD-1 expression and cytokine secretion of CD8^+^ CAR-T cells in the blood drawn at different time points (Fig. 6A). We observed that PD-1 was decreased in SFN-treated patients, whereas IFN-γ and IL-2 expression were increased (Fig. 6B–E), which indicated that SFN might have a potential therapeutic effect.

## Discussion

SFN has been shown to directly suppress tumor growth [20]. In this study, we investigated whether and how SFN regulates immune responses through its effect on CAR-T cells. We demonstrated that SFN effectively potentiated adaptive cellular therapy responses against tumor cells by inhibiting the PD-1/PD-L1 pathway, which indicated that SFN exhibited double benefits with CAR-T cells in mediating tumor regression.

A major limitation of CAR-T cells is the dysfunction and exhaustion of the infused T cells [33, 34]. PD-1 has been reported as a negative immune regulator related to T cell exhaustion in several studies [8]. Tumor-infiltrating CAR-T cells showed an increase in the expression of inhibitory receptors, including PD-1, consistent with low effector cytokine secretion upon engagement with PD-L1 [35]. Furthermore, PD-1 expression on HER2-specific CAR-T cells was increased after incubation with PD-L1^+^ tumor cells, whereas the effector functions of CAR-T cells were reversed by PD-1 blockade [36]. Our previous study showed that replacing the PD-1 glycosylated residue on CAR-T cells with the adenine base editor resulted in PD-1 suppression to augment CAR-T cell therapy [37]. In addition, we have reported that the anti-diabetic drug metformin could prevent the exhaustion of CD8^+^ T cells in patients by downregulating PD-1 expression [38]. Herein, we found that SFN elevated CAR-T cell antitumor function by impairing PD-1 expression through partial inhibition of the PI3K/AKT pathway. After SFN treatment, the killing ability and cytokine secretion of CAR-T cells were increased to some extent in vitro. Regardless of the method of administration, CAR-T cells from the SFN pre-treated group and the SFN exogenous injection group showed lower PD-1 expression and more potent antitumor function in vivo. Compared to treatment with CAR-T cells plus anti-PD-1, CAR-T cell therapy with exogenous SFN injection showed similar effects in a mouse model, which indicated that SFN might be additionally used for PD-1 blockade therapy.

Upregulation of PD-L1 on tumor cells suppresses CAR-T cell antitumor capacity after chronic antigen stimulation [39]. IFN-γ plays a role in inducing PD-L1 expression on tumor cells both in vitro and in the tumor microenvironment [12, 40]. We wondered whether SFN affected PD-L1 expression, in addition to its effect on PD-1 inhibition. In this study, we observed that SFN significantly downregulated the IFN-γ-mediated PD-L1 expression on tumor cells. In addition to SFN, other nutrients, including apigenin and curcumin, exhibit not only tumor cell suppression effect but also inhibit PD-L1 expression. Apigenin has been shown to restrict IFN-γ-induced PD-L1 expression on both human and mouse breast cancer cells by inhibiting STAT1 phosphorylation [41]. As for melanoma cells, apigenin and curcumin inhibited PD-L1 expression, besides tumor growth-suppressive and pro-apoptotic effects. Similar to the mechanism in breast cancer, both apigenin and curcumin impaired the phosphorylation of STAT1. In addition to tumor cells, apigenin and curcumin also decreased PD-L1 expression on DCs, which augmented T cell activity [42]. These findings revealed that apigenin and curcumin regulated PD-L1 through the IFN-γ-JAK-STAT signaling pathway at the transcriptional level. The mechanism by which IFN-γ controls PD-L1 expression has been well investigated. Upon IFN-γ stimulation, the activated JAK-STAT signaling pathway promotes the binding of the transcription factor IRF1 to the PD-L1 promoter, mediating the transcription of PD-L1 [10]. Although the IFN-γ signaling pathway plays a major role in the transcriptional regulation of PD-L1, other pathways also contribute to the control of PD-L1 expression.

PTMs, including ubiquitination, phosphorylation, glycosylation, methylation, and acetylation, play key roles in regulating protein biological processes, such as activity, degradation, and interaction [43]. Accumulating evidence has revealed that PTMs participate in PD-L1 regulation [32, 44, 45]. In our study, we found that PD-L1 expression was not significantly affected after SFN treatment at the transcriptional level, but was significantly downregulated at the protein level, which prompted us to hypothesize that PD-L1 undergoes PTMs that affect its stability. Finally, we found that ubiquitination was activated by SFN according to RNA-seq and GSEA analysis, and β-TrCP played a critical role in PD-L1 regulation. β-TrCP acts as an E3 ligase adaptor protein that has been reported to affect PD-L1 degradation [32]. Deng et al. found that inhibition of mTORC1/p70S6K resulted in the reduction of β-TrCP expression, which is involved in mediating PD-L1 degradation [46]. Interestingly, PD-L1 glycosylation antagonizes GSK3β binding, subsequently disrupting the β-TrCP interaction, which contributes to PD-L1 stability [47].

In Liang’s review, SFN inhibited T cell activation by increasing ROS level and decreasing GSH [48]. According to another paper from Liang, they treated T cells with SFN at 10 μM for 1 h and T cell activation was impaired, whereas a few studies found that SFN promoted the frequency and function of immune cells in vivo [49]. It is reported that the combination of SFN and doxorubicin inhibited MDSC expansion and enhanced antitumor activities of CD8^+^ T cell in mice model [50]. Thejass et al. demonstrated that SFN significantly enhanced the production of IL-2 and IFN- γ in NK cell in both normal and tumor-bearing mice model [51]. In our study, we observed the similar results in mice model and found that co-administration of SFN and CAR-T cell therapy restricted the tumor growth by promoting the cytotoxicity of CAR-T cells. Thus, the effect of SFN on immune cells is controversial. In order to explore the effect of different concentrations of SFN, CAR-T cells were treated with different concentration of SFN in in vitro assay. After treated with 5 μM, 10 μM, and 15 μM of SFN, CAR-T cells was cocultured with H322 cells for 6 h. Then, the specific lysis of tumor cells and CD107a expression on CAR-T cells were measured by flow cytometry. Tumor cell apoptosis and CD107a expression on CAR-T cells were increased significantly under the concentration of 15 μM (data not shown).

Since SFN displays dramatic outcomes in preclinical models, whether and how SFN affects human bodies should be taken into account. To date, several clinical trials have evaluated the effect of SFN in both healthy donors and patients with cancer [52]. A randomized, placebo-controlled, double-blind clinical study for safety of SFN extracts was conducted in healthy volunteers [53]. No significant adverse events were observed which provided evidence for the safety of SFN treatment. In addition, the efficacy of SFN in patients with cancer has also been investigated. In a double-blinded, randomized, placebo-controlled, multicenter clinical trial, 78 patients with biochemical recurrence after radical prostatectomy were randomly treated with 60 mg of SFN orally for 6 months, followed by 2 months without treatment. The median log PSA slopes were significantly lower, and PSA doubling time was longer in the SFN-treated group than in the placebo group [54]. The encouraging results from a randomized, double-blinded 3-arm parallel intervention for 12 months involved 49 participants diagnosed with low/intermediate-risk prostate cancer. The results showed that the consumption of broccoli was consistent with a decrease in the risk of cancer progression [55]. These findings demonstrated that patients with cancer could benefit from monotherapy with SFN-based preparations. However, whether patients could benefit from SFN treatment in combination with CAR-T cells is unclear. In this study, various patients with cancer received CAR-T therapy combined with oral SFN treatment. CAR-T cells from the SFN-treated group showed lower PD-1 expression and higher effector cytokine secretion compared with patients in the control group, which demonstrated that SFN promoted CAR-T cell effector function in clinical applications.

In our study, we found that SFN both decreased PD-1 expression on CAR-T cells and PD-L1 on tumor cells. Furthermore, CAR-T cell infusion combined with SFN administration showed a stronger ability against tumor growth in mice model, suggesting that this combination therapy may provide a strategy for the clinical application of immunotherapy. However, there are still several limitations in this study. Although we observed effect of SFN on patients with different type of cancer, the sample size we used was small with a relatively short observation time. The therapeutic effect of SFN for other tumors needs further verification. In addition, the antitumor mechanism of SFN in vivo remains unclear. For further study, we will expand the sample size of patients with solid tumor for observation of the safety and efficacy. Future in vivo study will be also needed to investigate the underlying mechanisms of SFN against tumor.

In conclusion, we reported that SFN not only potentiated CAR-T cell function by downregulating PD-1 expression, but also reduced PD-L1 expression by activating the ubiquitination-mediated proteolysis pathway, which indicated the double benefit of combination therapy in inducing tumor remission.

## Conclusions

In this study, we found that SFN regulated immune response through the PD-1/PD-L1 pathway. Further study showed that SFN downregulated PD-1 expression on CAR-T cells by inhibiting PI3K/AKT signaling pathway, and PD-L1 degradation was induced by SFN in a ubiquitination-dependent manner. In addition, SFN in combination with CAR-T cell treatment enhanced antitumor response both in mice and patients. Hence, SFN has a potential therapeutic effect in the design of combination therapies with CAR-T cells.

## Supplementary Information

**Additional file 1: Table S1.** Primers of genes.

**Additional file 2: Table S2.** Clinical characteristics of the enrolled patients.

**Additional file 3: Figure S1.** The quantitative analysis of Western blot data. **A** Relative expression of PD-1 protein level in control and SFN group. The quantitative analysis of PD-1, p-AKT, p-mTOR, p-S6, t-AKT, t-mTOR, t-S6 protein level in control group, SFN group, and PI3K/AKT inhibitor group. **B** Relative expression of PD-L1 protein level of control, IFN- γ and IFN- γ + SFN group in A549 and H322. **C** The quantitative analysis of β-TrCP protein level of control, IFN- γ and IFN- γ + SFN group in A549 and H322. **D** The quantitative analysis of β-TrCP protein level of siRNA transfection efficacy in A549 and H322 cells. **E** Relative expression of PD-L1 protein level was detected in the presence of IFN- γ or SFN after si-Scr or si-BTRC by western blot in A549 and H322 cells

## Data Availability

All data generated of analyzed during this study are included in this published article and its supplementary information files. The datasets generated and used in this study are available from the corresponding author on reasonable request.

## Abbreviations

CAR-T: Chimeric antigen receptor T cell
GSEA: Gene set enrichment analysis
IFN-γ: Interferon gamma
IHC: Immunohistochemistry
IL-2: Interleukin-2
PBMCs: Peripheral blood mononuclear cells
PD-1: Programmed cell death 1
PD-L1: Programmed cell death ligand 1
PTMs: Post-translational modifications
qRT-PCR: Quantitative real-time PCR
scFv: Single-chain fragment variable
SFN: Sulforaphane
siRNA: Short interfering RNA
TNF-α: Tumor necrosis factor alpha

## References

[1] June CH, Sadelain M (2018). Chimeric antigen receptor therapy. The New England journal of medicine..

[2] Tian Y, Li Y, Shao Y, Zhang Y (2020). Gene modification strategies for next-generation CAR T cells against solid cancers. J Hematol Oncol..

[3] Rafiq S, Hackett CS, Brentjens RJ (2020). Engineering strategies to overcome the current roadblocks in CAR T cell therapy. Nat Rev Clin Oncol..

[4] Larson RC, Maus MV (2021). Recent advances and discoveries in the mechanisms and functions of CAR T cells. Nature reviews Cancer..

[5] McLane LM, Abdel-Hakeem MS, Wherry EJ (2019). CD8 T cell exhaustion during chronic viral infection and cancer. Annu Rev Immunol..

[6] Hossain MA, Liu G, Dai B, Si Y, Yang Q, Wazir J, Birnbaumer L, Yang Y (2021). Reinvigorating exhausted CD8(+) cytotoxic T lymphocytes in the tumor microenvironment and current strategies in cancer immunotherapy. Med Res Rev..

[7] Chinai JM, Janakiram M, Chen F, Chen W, Kaplan M, Zang X (2015). New immunotherapies targeting the PD-1 pathway. Trends Pharmacol Sci..

[8] Baumeister SH, Freeman GJ, Dranoff G, Sharpe AH (2016). Coinhibitory pathways in immunotherapy for cancer. Annu Rev Immunol..

[9] Mandai M, Hamanishi J, Abiko K, Matsumura N, Baba T, Konishi I (2016). Dual faces of IFNgamma in cancer progression: a role of PD-L1 induction in the determination of pro- and antitumor immunity. Clin Cancer Res..

[10] Garcia-Diaz A, Shin DS, Moreno BH, Saco J, Escuin-Ordinas H, Rodriguez GA, Zaretsky JM, Sun L, Hugo W, Wang X, Parisi G, Saus CP, Torrejon DY, Graeber TG, Comin-Anduix B, Hu-Lieskovan S, Damoiseaux R, Lo RS, Ribas A (2017). Interferon receptor signaling pathways regulating PD-L1 and PD-L2 expression. Cell Rep..

[11] Pistillo MP, Carosio R, Banelli B, Morabito A, Mastracci L, Ferro P, Varesano S, Venè R, Poggi A, Roncella S (2020). IFN-gamma upregulates membranous and soluble PD-L1 in mesothelioma cells: potential implications for the clinical response to PD-1/PD-L1 blockade. Cell Mol Immunol..

[12] Abiko K, Matsumura N, Hamanishi J, Horikawa N, Murakami R, Yamaguchi K, Yoshioka Y, Baba T, Konishi I, Mandai M (2015). IFN-gamma from lymphocytes induces PD-L1 expression and promotes progression of ovarian cancer. Br J Cancer..

[13] Callahan MK, Kluger H, Postow MA, Segal NH, Lesokhin A, Atkins MB, Kirkwood JM, Krishnan S, Bhore R, Horak C, Wolchok JD, Sznol M (2018). Nivolumab plus ipilimumab in patients with advanced melanoma: updated survival, response, and safety data in a Phase I dose-escalation study. Journal of clinical oncology : official journal of the American Society of Clinical Oncology..

[14] Hellmann MD, Ciuleanu TE, Pluzanski A, Lee JS, Otterson GA, Audigier-Valette C, Minenza E, Linardou H, Burgers S, Salman P, Borghaei H, Ramalingam SS, Brahmer J, Reck M, O’Byrne KJ, Geese WJ, Green G, Chang H, Szustakowski J, Bhagavatheeswaran P, Healey D, Fu Y, Nathan F, Paz-Ares L (2018). Nivolumab plus ipilimumab in lung cancer with a high tumor mutational burden. The New England journal of medicine..

[15] Bellmunt J, de Wit R, Vaughn DJ, Fradet Y, Lee JL, Fong L, Vogelzang NJ, Climent MA, Petrylak DP, Choueiri TK, Necchi A, Gerritsen W, Gurney H, Quinn DI, Culine S, Sternberg CN, Mai Y, Poehlein CH, Perini RF, Bajorin DF, KEYNOTE-045 Investigators (2017). Pembrolizumab as second-line therapy for advanced urothelial carcinoma. The New England journal of medicine..

[16] Ribas A, Wolchok JD (2018). Cancer immunotherapy using checkpoint blockade. Science (New York, NY).

[17] Bagchi S, Yuan R, Engleman EG (2021). Immune checkpoint inhibitors for the treatment of cancer: clinical impact and mechanisms of response and resistance. Annu Rev Pathol..

[18] Palliyaguru DL, Yuan JM, Kensler TW, Fahey JW (2018). Isothiocyanates: translating the power of plants to people. Mol Nutr Food Res..

[19] Zhang Y, Talalay P, Cho CG, Posner GH (1992). A major inducer of anticarcinogenic protective enzymes from broccoli: isolation and elucidation of structure. Proceedings of the National Academy of Sciences of the United States of America..

[20] Bayat Mokhtari R, Baluch N, Homayouni TS, Morgatskaya E, Kumar S, Kazemi P, Yeger H (2018). The role of sulforaphane in cancer chemoprevention and health benefits: a mini-review. J Cell Commun Signal..

[21] Lenzi M, Fimognari C, Hrelia P (2014). Sulforaphane as a promising molecule for fighting cancer. Cancer Treat Res..

[22] Clarke JD, Dashwood RH, Ho E (2008). Multi-targeted prevention of cancer by sulforaphane. Cancer Lett..

[23] Briones-Herrera A, Eugenio-Perez D, Reyes-Ocampo JG, Rivera-Mancia S, Pedraza-Chaverri J (2018). New highlights on the health-improving effects of sulforaphane. Food Funct..

[24] Park HS, Han MH, Kim GY, Moon SK, Kim WJ, Hwang HJ, Park KY, Choi YH (2014). Sulforaphane induces reactive oxygen species-mediated mitotic arrest and subsequent apoptosis in human bladder cancer 5637 cells. Food Chem Toxicol..

[25] Li Y, Zhang T, Korkaya H, Liu S, Lee HF, Newman B, Yu Y, Clouthier SG, Schwartz SJ, Wicha MS, Sun D (2010). Sulforaphane, a dietary component of broccoli/broccoli sprouts, inhibits breast cancer stem cells. Clin Cancer Res..

[26] Burnett JP, Lim G, Li Y, Shah RB, Lim R, Paholak HJ, McDermott SP, Sun L, Tsume Y, Bai S, Wicha MS, Sun D, Zhang T (2017). Sulforaphane enhances the anticancer activity of taxanes against triple negative breast cancer by killing cancer stem cells. Cancer Lett..

[27] Shih YL, Wu LY, Lee CH, Chen YL, Hsueh SC, Lu HF (2016). Sulforaphane promotes immune responses in a WEHI3induced leukemia mouse model through enhanced phagocytosis of macrophages and natural killer cell activities in vivo. Mol Med Rep..

[28] Geisel J, Bruck J, Glocova I, Dengler K, Sinnberg T, Rothfuss O (2014). Sulforaphane protects from T cell-mediated autoimmune disease by inhibition of IL-23 and IL-12 in dendritic cells. J Immunol..

[29] Lanitis E, Poussin M, Hagemann IS, Coukos G, Sandaltzopoulos R, Scholler N, Powell DJ (2012). Redirected antitumor activity of primary human lymphocytes transduced with a fully human anti-mesothelin chimeric receptor. Mol Ther..

[30] Subramanian A, Tamayo P, Mootha VK, Mukherjee S, Ebert BL, Gillette MA, Paulovich A, Pomeroy SL, Golub TR, Lander ES, Mesirov JP (2005). Gene set enrichment analysis: a knowledge-based approach for interpreting genome-wide expression profiles. Proceedings of the National Academy of Sciences of the United States of America..

[31] Herrero-Sanchez MC, Rodriguez-Serrano C, Almeida J, San Segundo L, Inoges S, Santos-Briz A (2016). Targeting of PI3K/AKT/mTOR pathway to inhibit T cell activation and prevent graft-versus-host disease development. J Hematol Oncol..

[32] Zhang J, Dang F, Ren J, Wei W (2018). Biochemical aspects of PD-L1 regulation in cancer immunotherapy. Trends Biochem Sci..

[33] Poorebrahim M, Melief J, Pico de Coana Y, LW S, Cid-Arregui A, Kiessling R (2021). Counteracting CAR T cell dysfunction. Oncogene..

[34] Zhang Z, Liu S, Zhang B, Qiao L, Zhang Y, Zhang Y (2020). T Cell dysfunction and exhaustion in cancer. Front Cell Dev Biol..

[35] Cherkassky L, Morello A, Villena-Vargas J, Feng Y, Dimitrov DS, Jones DR, Sadelain M, Adusumilli PS (2016). Human CAR T cells with cell-intrinsic PD-1 checkpoint blockade resist tumor-mediated inhibition. The Journal of clinical investigation..

[36] John LB, Devaud C, Duong CP, Yong CS, Beavis PA, Haynes NM (2013). Anti-PD-1 antibody therapy potently enhances the eradication of established tumors by gene-modified T cells. Clin Cancer Res..

[37] Shi X, Zhang D, Li F, Zhang Z, Wang S, Xuan Y, Ping Y, Zhang Y (2019). Targeting glycosylation of PD-1 to enhance CAR-T cell cytotoxicity. J Hematol Oncol..

[38] Zhang Z, Li F, Tian Y, Cao L, Gao Q, Zhang C, Zhang K, Shen C, Ping Y, Maimela NR, Wang L, Zhang B, Zhang Y (2020). Metformin enhances the antitumor activity of CD8(+) T lymphocytes via the AMPK-miR-107-Eomes-PD-1 pathway. J Immunol..

[39] Grosser R, Cherkassky L, Chintala N, Adusumilli PS (2019). Combination immunotherapy with CAR T cells and checkpoint blockade for the treatment of solid tumors. Cancer Cell..

[40] Abiko K, Mandai M, Hamanishi J, Yoshioka Y, Matsumura N, Baba T, Yamaguchi K, Murakami R, Yamamoto A, Kharma B, Kosaka K, Konishi I (2013). PD-L1 on tumor cells is induced in ascites and promotes peritoneal dissemination of ovarian cancer through CTL dysfunction. Clin Cancer Res..

[41] Coombs MR, Harrison ME, Hoskin DW (2016). Apigenin inhibits the inducible expression of programmed death ligand 1 by human and mouse mammary carcinoma cells. Cancer Lett..

[42] Xu L, Zhang Y, Tian K, Chen X, Zhang R, Mu X, Wu Y, Wang D, Wang S, Liu F, Wang T, Zhang J, Liu S, Zhang Y, Tu C, Liu H (2018). Apigenin suppresses PD-L1 expression in melanoma and host dendritic cells to elicit synergistic therapeutic effects. J Exp Clin Cancer Res..

[43] Hattori T, Koide S (2018). Next-generation antibodies for post-translational modifications. Curr Opin Struct Biol..

[44] Gao Y, Nihira NT, Bu X, Chu C, Zhang J, Kolodziejczyk A, Fan Y, Chan NT, Ma L, Liu J, Wang D, Dai X, Liu H, Ono M, Nakanishi A, Inuzuka H, North BJ, Huang YH, Sharma S, Geng Y, Xu W, Liu XS, Li L, Miki Y, Sicinski P, Freeman GJ, Wei W (2020). Acetylation-dependent regulation of PD-L1 nuclear translocation dictates the efficacy of anti-PD-1 immunotherapy. Nat Cell Biol..

[45] Xiang J, Zhang N, Sun H, Su L, Zhang C, Xu H, Feng J, Wang M, Chen J, Liu L, Shan J, Shen J, Yang Z, Wang G, Zhou H, Prieto J, Ávila MA, Liu C, Qian C (2020). Disruption of SIRT7 increases the efficacy of checkpoint inhibitor via MEF2D regulation of programmed cell death 1 ligand 1 in hepatocellular carcinoma cells. Gastroenterology..

[46] Deng L, Qian G, Zhang S, Zheng H, Fan S, Lesinski GB, Owonikoko TK, Ramalingam SS, Sun SY (2019). Inhibition of mTOR complex 1/p70 S6 kinase signaling elevates PD-L1 levels in human cancer cells through enhancing protein stabilization accompanied with enhanced beta-TrCP degradation. Oncogene..

[47] Li CW, Lim SO, Xia W, Lee HH, Chan LC, Kuo CW, Khoo KH, Chang SS, Cha JH, Kim T, Hsu JL, Wu Y, Hsu JM, Yamaguchi H, Ding Q, Wang Y, Yao J, Lee CC, Wu HJ, Sahin AA, Allison JP, Yu D, Hortobagyi GN, Hung MC (2016). Glycosylation and stabilization of programmed death ligand-1 suppresses T-cell activity. Nature communications..

[48] Liang J, Hansch GM, Hubner K, Samstag Y (2019). Sulforaphane as anticancer agent: a double-edged sword? Tricky balance between effects on tumor cells and immune cells. Adv Biol Regul..

[49] Liang J, Jahraus B, Balta E, Ziegler JD, Hubner K, Blank N (2018). Sulforaphane inhibits inflammatory responses of primary human T-cells by increasing ROS and depleting glutathione. Frontiers in immunology..

[50] Rong Y, Huang L, Yi K, Chen H, Liu S, Zhang W, Yuan C, Song X, Wang F (2020). Co-administration of sulforaphane and doxorubicin attenuates breast cancer growth by preventing the accumulation of myeloid-derived suppressor cells. Cancer Lett..

[51] Thejass P, Kuttan G (2006). Augmentation of natural killer cell and antibody-dependent cellular cytotoxicity in BALB/c mice by sulforaphane, a naturally occurring isothiocyanate from broccoli through enhanced production of cytokines IL-2 and IFN-gamma. Immunopharmacol Immunotoxicol..

[52] Yagishita Y, Fahey JW, Dinkova-Kostova AT, Kensler TW. Broccoli or sulforaphane: is it the source or dose that matters? Molecules. 2019;24(19):3593. 10.3390/molecules24193593 PMC6804255 31590459

[53] Shapiro TA, Fahey JW, Dinkova-Kostova AT, Holtzclaw WD, Stephenson KK, Wade KL, Ye L, Talalay P (2006). Safety, tolerance, and metabolism of broccoli sprout glucosinolates and isothiocyanates: a clinical phase I study. Nutr Cancer..

[54] Cipolla BG, Mandron E, Lefort JM, Coadou Y, Della Negra E, Corbel L, le Scodan R, Azzouzi AR, Mottet N (2015). Effect of sulforaphane in men with biochemical recurrence after radical prostatectomy. Cancer Prev Res (Phila)..

[55] Traka MH, Melchini A, Coode-Bate J, Al Kadhi O, Saha S, Defernez M (2019). Transcriptional changes in prostate of men on active surveillance after a 12-mo glucoraphanin-rich broccoli intervention-results from the Effect of Sulforaphane on prostate CAncer PrEvention (ESCAPE) randomized controlled trial. Am J Clin Nutr..

