# Supplementary material for: Sulforaphane enhances the antitumor response of chimeric antigen receptor T cells by regulating PD-1/PD-L1 pathway
Source: BMC Med. 2021 Nov 25;19:283. doi: 10.1186/s12916-021-02161-8 (PMC8614004; doi:10.1186/s12916-021-02161-8)
Supplement: Supplementary file 1 — Additional file 1: Table S1. Primers of genes. [file 12916_2021_2161_MOESM1_ESM.docx]

**Supplementary Table 1. Primers of genes.**

| Primer | Forward | Reverse |
| --- | --- | --- |
| GAPDH | 5’-CTGGGCTACACTGAGCACC-3’ | 5’-AAGTGGTCGTTGAGGGCAATG-3’ |
| PD-L1 | 5’-TGGCATTTGCTGAACGCATTT-3’ | 5’-TGCAGCCAGGTCTAATTGTTTT-3’ |
| BTRC | 5’-TGCCCAAGCAACGGAAACT-3’ | 5’-GCCCATGTTGGTAATGACACA-3’ |
