# Supplementary material for: Sulforaphane enhances the antitumor response of chimeric antigen receptor T cells by regulating PD-1/PD-L1 pathway
Source: BMC Med. 2021 Nov 25;19:283. doi: 10.1186/s12916-021-02161-8 (PMC8614004; doi:10.1186/s12916-021-02161-8)
Supplement: Supplementary file 2 — Additional file 2: Table S2. Clinical characteristics of the enrolled patients. [file 12916_2021_2161_MOESM2_ESM.docx]

**Supplementary Table 2. Clinical characteristics of the enrolled patients.**

| **Characteristics** | **SFN treated patients** | | | | **Non-SFN treated patients** | | | |
| --- | --- | --- | --- | --- | --- | --- | --- | --- |
|  | **SFN-1** | **SFN-2** | **SFN-3** | **SFN-4** | **Control-1** | **Control-2** | **Control-3** | **Control-4** |
| **Gender** | Male | Male | Female | Male | Male | Female | Male | Female |
| **Age (year)** | 15 | 13 | 67 | 56 | 15 | 53 | 45 | 54 |
| **Diagnosis** | B-ALL/LBL | ALL | HCC | RCCC | B-ALL/LBL | DLBCL | DLBCL | DLBCL |
| **Stage*** | IV | - | III | IV | IV | IV | IV | IV |
| **Treatment** | FC | FC | CTX | CTX | FC | FC | FC | FC |

*The stage of HCC and RCCC was based on TNM staging system. The stage of B-ALL/LBL and DLBCL was based on Ann Arbor staging system.

B-ALL/LBL: B-acute lymphoblastic leukemia/lymphoblastic lymphoma; ALL: acute lymphocytic leukemia; HCC: hepatocellular carcinoma; RCCC: renal clear cell carcinoma; DLBCL: diffuse large B cell lymphoma; FC: Fludarabine and Cyclophosphamide; CTX: Cyclophosphamide.
